# ‘I wasn’t on the front line *per se*, but I was part of health care’: Contributions and experiences of ancillary staff in care homes in England during the COVID-19 pandemic

**DOI:** 10.1177/13558196241246178

**Published:** 2024-04-20

**Authors:** Olivia Luijnenburg, Kritika Samsi, Ian Kessler, Caroline Norrie, Stephen Martineau, Jill Manthorpe

**Affiliations:** NIHR Health and Social Care Workforce Research Unit, 4616 King’s College London, London, UK

**Keywords:** care home, ancillary staff, COVID-19

## Abstract

**Objectives:**

Ancillary staff – cleaning, catering, housekeeping and laundry workers – play a crucial role in care homes, by promoting infection control, food preparation and hygiene, and contributing to the care home environment. This study sought to understand the experiences of ancillary staff working in English care homes during the COVID-19 pandemic. The results will inform policy makers, employers, care home managers and others, both in England and overseas, as how to best support the ancillary workforce.

**Methods:**

Between March and August 2021, video and telephone interviews were conducted with those working or living in care homes in England. Participants comprised ancillary staff (*n* = 38), care home managers (*n* = 8), care home residents’ family members and friends (*n* = 7), human resource managers (*n* = 5) and care home residents (*n* = 5).

**Results:**

Ancillary staff often had increased responsibilities and contributed to pandemic efforts by changing working practices, routines and job roles with the aim of supporting residents and other staff. Teamwork, underpinned by strong leadership, helped ancillary staff feel supported.

**Conclusions:**

Ancillary staff should be better recognised as being central to care home care. They are essential workers helping to keep residents safe and well.

## Introduction

Care home workers are often overlooked in general workforce research,^1^ with the cooking, cleaning and housekeeping workforce (hereafter called ancillary staff) in care homes especially under-represented. The contributions of ancillary staff are vital to the running of the care home and enhancing the wellbeing of care home residents – for example, keeping the facility clean and comfortable, cooking meals and providing company. During the COVID-19 pandemic, ancillary workers in care homes provided additional and deeper cleaning, managed new infection control measures, as well as kept operations running with laundry, meal preparation and serving. However, beyond anecdotes and news reports,^2^ this contribution has been overlooked in more formal commentaries on the management of the pandemic. Global accounts from March 2020, curated by the International Long-Term Care Network on management of the crisis in social care, generally fail to mention ancillary workers. This workforce is not often heard from directly, and their experiences are rarely studied. In this present qualitative study, ancillary staff, their managers/supervisors and care home residents and their family members were interviewed about the experiences of ancillary staff working in care homes during the COVID-19 pandemic.

While ancillary staff include a range of roles – such as cooks, cleaning staff, housekeepers and laundry staff – the term is used in this paper to distinguish this group from other frontline care workers, care home nurses and activity co-ordinators. However, there are similarities in that both are largely female, work part-time on or near the United Kingdom (UK) national living wage,^3^ and many are migrants and/or from minority ethnic groups.^4^ Several studies have considered the pandemic related experiences of frontline care home workers,^5,6^ but there is a lack of literature investigating the specific contributions and experiences of ancillary staff in English care homes during the COVID-19 pandemic. This includes the mechanisms participants reported as supporting them, and how they felt they could be supported better, as the sector resets from the worst of the pandemic and learns to live with the virus.

In the UK, a distinction is made between health care and social care. Adult social care pertains to helping people that are older, living with a disability, or physical or mental illness to live as independently as possible.^7^ Care homes fall under the adult social care policy, and it would be most comparable with what is called ‘long-term care’ in other countries.^8^ Local authorities are responsible for determining the care needs of individuals and therefore the funding they are entitled to. Most care homes are run by independent care providers and are usually for profit.^7^ There are two main types of care homes in the UK: residential care homes and nursing homes. In residential care homes, ‘home-style’ live-in accommodation is provided, and residents have help with personal care such as washing, dressing, taking medicines and going to the toilet. In nursing homes, additional to the care one receives in a residential care home, medical care is available.^9^ This research recruited participants from both types of care homes for older people.

In the UK, the first guidance for reducing the risk of transmission of COVID-19 in residential care settings came out on March 13, 2020. The first lockdown was announced on March 23 and in May 2020 a care home support package of £600 million was announced. In June 2021, legislation was introduced banning the movement of staff between care settings.^10^ There was an excess of about 27,000 deaths from mid-March to June 2020 in care homes, which was 45% of all excess deaths nationally.^11^

Although data collection for this study occurred in between the third and fourth lockdown in the UK, the data reflects experiences throughout the pandemic. This is because the interviews included questions about the whole period of the pandemic, from before the first lockdown to all three lockdowns. Although our focus point is the ancillary workforce in English care homes, our findings will be of interest to care home providers internationally due to the scarcity of research about and guidelines for the ancillary workforce in care homes.

## Methods

### Study design and recruitment

This 10-month study lasted from January 1 to October 31, 2021. We wanted to capture ancillary staff’s experiences by speaking to them directly, and also to gain key informants’ views of the ancillary experience – care home managers, human resource (HR) managers in larger care home chains, care home residents and their relatives. Amongst the ancillary workers, we recruited chefs, cleaners, laundry staff, as well as those in supervisory roles, to understand their experiences. Including the views of managers, residents and their relatives result in a more holistic perspective of the value of this workforce and how their roles might have changed during the pandemic. The Public and Patient Involvement Group at the authors’ research unit advised the research team to include residents and their relatives in the study, to gain insight into daily life in the care home and the care home culture. Thus, their views on the experiences of ancillary staff were deemed a highly valuable addition, and this was indeed what we found during data analysis. (The Public and Patient Involvement Group consists of people with lived experience of using health and social care, including carers, people living with long-term conditions, and members of the public interested in health and social care. The group discusses and contributes to the design of the unit’s projects.)

Through existing networks, word of mouth and social media, we circulated study advertising materials. The NIHR Clinical Research Network and the ENRICH network of care homes helped publicise the study. We also adopted ‘snowballing sampling’, similarly referred to as ‘chain referral sampling’,^12^ asking participants to recommend friends or colleagues working in similar roles. An advisory group supported this study (comprising six members, including residents’ family members, former carers, care home managers and a member of a staff representative group) and supported recruitment by circulating study information to their networks. The research team met online with the advisory group before and after data collection in February and September 2021 and discussed emerging findings. Furthermore, the group helped in advising if data collected could be affected by poor recall due to the time passed since the first lockdown and the extreme circumstances during this time. All participating ancillary staff, residents and relatives were offered a £20 voucher in appreciation of their time.

### Data collection

Data collection took place between March and August 2021 – between the second and third national UK lockdowns as restrictions were eased, although less so for care homes. Some care homes were allowing visitors on strict conditions, where other care homes were in lockdown due to an outbreak. All interested participants were interviewed at a mutually convenient time. All but one of the interviews were conducted in English (the other being in Hindi, conducted by [anonymised]). In-depth interview topic guides were designed to ask about experiences of working in a care home during the pandemic, what the role of ancillary staff was during this time, what support had been provided and what gaps in support were identified. Questions were tailored according to the participant group. Interviews were conducted remotely – either over video-conferencing facility (such as Zoom or Teams) or via telephone – and lasted between 25 and 65 min. Interviews were recorded and transcribed. Brief demographic details were also collected.

### Data analysis

All interview transcripts were subjected to thematic analysis and scrutinised for consistencies or trends.^13^ The qualitative data analysis software NVivo (version 1.6.1) supported the analytical process. Line-by-line coding was conducted of first transcripts, and a broad coding framework was developed. Three members of the research team used the NVivo file in turns to go through codes already made and add any new codes (with initials of the researcher behind the code). This framework was iteratively developed as sub-themes were identified. Discussions with the wider research team, including the advisory group, enabled consensus to be reached regarding themes generated. The online supplement details how feedback from stakeholder workshops was incorporated into finalising themes and good practice advice. The research team were of mixed gender, age and ethnicity and included care home and HR researchers.

## Participants

Sixty-three semi-structured video and telephone interviews were conducted. These were with ancillary staff (*n* = 38), care home managers (*n* = 8), HR managers (*n* = 5), care home residents (*n* = 5) and their family members (*n* = 7) in care homes in England. Table 1 provides a breakdown of participants’ roles.

**Table 1.** Participants’ roles (*n* = 63).

Participant group	Number
Ancillary staff, comprising	38
- Catering or kitchen staff (14)	
- Housekeeping staff (12)	
- Cleaners (9)	
-Hospitality/ancillary staff manager (3)^a^	
Care home managers	8
Relatives/friends of care home residents	7
Human resource managers	5
Care home residents	5

^a^The titles given are as participants described themselves. One participant described themselves as a hospitality manager and had the same role as the ancillary staff managers.

Table 2 presents demographic categories of the ancillary workforce participants, including ethnicity, gender and age. Most were White British, female, and aged 41 and over.

**Table 2.** Demographic details of ancillary worker participants (*n* = 38).

Demographic category	Sub-category	Number
Ethnicity	British White	23
	British Asian (Indian/Pakistani)	8
	Black African	2
	Black Caribbean	2
	Filipino	2
	Declined to answer	1
Gender	Female	27
	Male	10
	Declined to answer	1
Age	18–29	3
	30–40	7
	41–50	12
	51–60	13
	>60	3

## Results

An overarching theme identified in all transcripts related to the significant contributions of ancillary staff during the pandemic, and the structures that supported these. Below, we describe this theme as reflected in three sub-themes: (1) changed patterns of work, (2) organisational support and (3) feeling rewarded and recognised.

### Changed patterns of work

Changed patterns of work were described by many participants, reflecting the contributions ancillary staff made and the flexibility they demonstrated.

#### Increase in cleaning procedures

Ancillary staff participants reported undertaking more cleaning tasks during the pandemic. These included cleaning ‘touch points’ (for example, light switches, rails and lift buttons) at least five times a day. Deployment of staff to separate sections of the care home and ensuring separate meal breaks from each other occurred in most homes to sustain social distancing.

In homes or in sections of homes without COVID-19 positive-tested residents, all staff wore masks, as mask-wearing was mandatory, and some mentioned wearing gloves and aprons, while others would wash hands regularly. If there was a COVID-19 outbreak, cleaners had to don full Personal Protective Equipment (PPE) when cleaning the rooms of residents who tested positive and had to dispose of this PPE after each room visit. Many participants that were responsible for laundry reported that the laundry of people with COVID-19 was put in colour-coded bags and people working in the laundry had to wear full PPE.

Care home and HR managers reported that new equipment was introduced, which ancillary staff had to be trained how to use:

We bought a fogging machine, so it was getting them trained on all the new products that we were bringing into the building and what to use on what surfaces and things like that. (HR manager 5)

This also relates to the upskilling of staff, which is discussed further under the theme of organisational support.

In kitchens, rigorous cleaning procedures were usually already in place, but in some care homes, groceries were being effectively quarantined for 72 hours before being taken into the kitchen. Changes in terms of workload and new equipment or procedures appeared more pronounced for cleaning and housekeeping staff compared to kitchen staff. Furthermore, kitchen staff remained in the kitchens to maintain social distancing and reduce mixing.

#### ‘We did what we had to do’

A strong sense of teamwork and a ‘can do’ attitude was described by many ancillary, management, and resident and relative participants. Instances were described of the whole care home staff team coming together, with a common goal of keeping all residents safe:

What I’m proud of is how everybody came together as a team and focused on helping to keep people safe. That was the senior management team down to all the managers. If there was one team that went off and had to self-isolate because of the infection, there would always be some senior managers here in another team. And that we were here, we rolled up our sleeves and we were dropping meals off to people who were isolating in their flats and in their rooms. We were covering shifts; we did what we had to do. (Manager 4)

Some managers arranged transport for staff to avoid them relying on public transport. Others set up a car sharing scheme and changed shift patterns so staff could drive together. One participant organised taxis or other private transport for staff. Some staff started walking or cycling to work.

#### Commitment and personal challenges

Some participants described circumstances that made it difficult for them to continue work, including parenting or other family responsibilities, necessitating their taking time off work or needing more flexible shift patterns. Some of these decisions had challenging financial implications:

I must admit, when it first started, I was a bit worried about it. And they did say at the time that if you can’t work obviously, you can stay home, but you won’t get paid. So, we couldn’t afford to stay home. We all had to work through it. But, luckily, we got through it. (Housekeeper 1)

One participant, who had a heightened risk of COVID-19 infection, initially felt supported by their manager, but her removal from all areas was not sustainable and she agreed to help out:

I’ve got COPD [chronic obstructive pulmonary disease], so I was really panicky about having to go and clean the rooms. So [my manager] sort of said you don’t need to do them basically. But it’s hard because … we’ve had [COVID-19] on all floors in the end. So, I have to go and clean the COVID rooms. Not as much as the other girls. But I’ve had to go and do them. (Cleaner 2)

Many care home staff recounted how the responsibility of keeping residents safe made them feel anxious. This could manifest itself outside of work:

A couple of times driving into the supermarket, if I saw the car park was full, I started getting quite anxious and palpitations. And I've never been like that before. And I think it's just with knowing where I worked. I knew how healthy I had to be … It would make me quite worried, but I'd still go through and do it. (Chef 1)

A resident confirmed such thoughts, and mentioned that working as a cleaner in a care home is different from in a private home:

Working for a home is not the same as working in a private house. They’ve got certain standards to meet and if they don’t meet them, they won’t be retained. (Resident 2)

One relative who volunteered in their relative’s care home during the pandemic talked of staff camaraderie. The participant observed a strong sense of personal responsibility among individual workers towards keeping residents safe:

Staff were taking it really seriously because nobody wanted to be that person who would bring in COVID. I remember myself thinking: ‘It can’t be me that brings COVID in when I’m volunteering’. (Relative 1)

This participant was the only relative that volunteered as a family member in a care home.

### Organisational support

Ancillary staff participants felt they needed, and some felt they were offered, adequate support to navigate the emotional challenges of the pandemic. This support took the form of support from management, colleagues and upskilling.

#### Support from management

Meaningful support from managers was described by some ancillary staff participants as important to their wellbeing. One manager immediately responded to needs for equipment or supplies:

As soon as I asked for anything, you know, if I’m low on the clinical wipes, [the manager] is there, he’ll order them for me. PPE, I say ‘right we need this’, it’s there. So absolutely 100%, totally backed by [the manager]. (Housekeeper 3)

On the other hand, some managers reported a lack of governmental understanding on how care homes operate. They felt the guidance they received often did not reflect what was necessary or possible for care home staff:

We lost 90% of our staff [off work with COVID-19, isolating, etc.], and at one point, it was … just me, one cleaner, and a cook, and agency staff. … [The NHS and local authority were asking us] how are you dealing with clinical waste? How are you covering your staff? How are you doing XYZ? But they were throwing it all back to the care home owners and just saying ‘Look, sort this out … do this big report for us’. And that was just crazy. And we had to sort of reinvent the wheel for them, which was all deeply frustrating because it showed a lack of trust [and] we just didn't have any manpower to do all this stuff. (HR manager 1)

Although most ancillary workers viewed communication from their managers as adequate, some felt they were not always brought up to date with the latest information on cleaning, testing or other changes. Only one of the ancillary staff participants shared dissatisfaction with management support:

I have to say you know at the care home, for a while, I thought I'm putting my life at risk by working there, but there was no other way you know? I spoke to the care home manager and she didn't really say much but that “I'd be expecting you to come in each week during this difficult period.” So there was no other way so I just kept reporting in and just doing my duties. (Cleaner 4)

It is remarkable that this was the only participant that shared negative experiences. This will be considered further in the Discussion section below.

#### Collegial support

Many staff described turning to each other for support during the pandemic, more than they had prior to COVID-19.

Group support developed organically, using WhatsApp phone groups or email chains, and some staff described ensuring that colleagues who were shielding or on furlough (paid but not working) could still feel part of the team. Most care home managers mentioned that a mentor or ‘buddy’ scheme was in place, where ancillary staff had a colleague ‘on the floor’ to support them. Managers’ ‘open door policy’ was repeatedly mentioned, direct communication was possible, and trust and confidentiality promoted. Managers mentioned that some of the changes introduced to support staff through the pandemic have been retained:

You’re a bit more aware of other people’s emotional needs, rather than coming in and doing your day job and worrying about your time and energies, we’ve all really looked out for each other … I do a wellbeing Wednesday email, which I send out every week. (HR manager 2)

#### Upskilling ancillary staff

Managers’ support also included offering new development opportunities to ancillary staff during the pandemic, sometimes as a necessity:

We’ve tried to make sure that if we did have people go down [with the virus], then other people could step into their shoes and know how to do their job. That’s helped in a huge way. And in respect of trying to empower people to take on different roles, really … [This went] right across the board. Kitchen [staff] were learning about outside on the floor, and, each person, they spent time in each area, so if people did go off sick … we didn’t have to use agency [locum staff]. (HR manager 5)

Managers and HR managers reported different experiences with hiring staff from agencies or not. One care home manager said they did not want to have agency staff due to the potential for cross-contamination between different areas in the home or different care homes. They filled any gaps of ancillary staff with other staff doing overtime or covering these shifts. But other managers said they did get agency staff in, although they tried to always get the same staff in to minimise any spread of infection.

#### Mental health support

Anxiety was high particularly in the early months of the pandemic, causing some care home staff participants to take time off work. Several managers mentioned how their workforce had sometimes halved in number during the early lockdowns or during a COVID-19 outbreak. Some homes held small ceremonies or commemorations of residents who had died during the pandemic. However, this practice was not widespread, and some ancillary staff were still grieving:

One of my residents caught [the virus]. When I had it, she too had it. I never knew that she had it. So when I recovered [and came back to work, I found out] she died. I wasn’t there when she died. Yeah, so when I resumed work, I was crying … all those people gone. (Housekeeper 7)

Some mental health support was mentioned. One manager had set up a 24-hour helpline during COVID-19 which was accessible to ancillary staff for emotional support. Ancillary staff participants also talked about receiving weekly email mental health bulletins from supervisors and feeling they could talk with their colleagues or manager. Most participating ancillary staff reported they had coped well and felt supported.

### Feeling rewarded and recognised

Several ancillary workers raised the importance of rewards and recognition for what they considered difficult and skilled jobs.

#### Rewards

Managers were aware of the demands the pandemic had placed on all staff, including ancillary workers. One said it became harder to maintain staff morale as the pandemic wore on and energy started to flag:

It’s been very emotionally and physically hard … Especially with domestic and housekeeping: they’ve had to pick up extra [work] because of extra cleaning that we’ve been doing. But also the mental sort of side – we’re making sure that they’re having regular breaks, but … I don’t feel they get that full recharge [that] you would normally do on a proper holiday. So, keeping people going has probably been the hardest thing. (Manager 2)

Some managers and supervisors provided non-monetary rewards to recognise ancillary staff’s contributions during the pandemic, including extra breaks, small treats to all staff, food and drink:

If [someone is working] longer hours, [we] offer them tea, coffee, beverages … [The] coffee machine is making a different impact … they can have lattés any time. They can have food, [which we offer] to them, so they can relax, don’t need to think about when they go home … So, we’ve been giving different benefits and recognition, rather than giving funding directly (Hospitality manager 1)

#### Intrinsic job satisfaction

Most ancillary staff participant accounts of the pandemic were positive about their work during it, the contributions they felt they had made, and were content with the support they received. Cleaners who had meaningful interactions with care home residents found this improved their job satisfaction. Several ancillary staff participants felt lucky to be able to work, as it gave them a sense of purpose:

It was nice to have a reason to go out. It was nice to know that you had a role and a purpose, and you were supporting those that couldn’t support themselves. So, I know I wasn’t on the front line per se, but I was part of health care - well, indirect health care, because I was in the kitchen. But of course, good nutrition is important. (Kitchen assistant 1)

All kitchen staff participants mentioned being prevented from going into residents’ living space, whereas cleaning and laundry staff would have close contact with residents and enter their rooms. This was mainly due to social distancing guidelines and practices. Some kitchen staff did not like this reduced engagement with residents and other staff, but others felt it was better to focus on their kitchen work rather than on direct care for residents.

#### Recognition

Some ancillary staff believed recognition of their work had increased, both from colleagues and the public, during the pandemic. Others thought there was never enough recognition for their physically and mentally demanding work, and that sometimes only direct care workers were recognised in initiatives:

But it's just so heartbreaking that when they have employee of the month, we never get recognised, it’s always the carers (care workers). You know, we do a good job. If it wasn’t for us cleaning, you know, they could have had this virus in the home. But we kept on top of all the cleaning. And we don’t get recognised, which is very unfair. (Housekeeper 1)

Ancillary staff perceived they had more recognition during the pandemic due to their ‘key worker status’. But one relative observed that this new status may have been a double-edged sword as it required people to put themselves in the line of danger during the pandemic:

Whilst it’s nice that they are classed as key workers, I felt there was a bit of an underhand thing: that if you were a key worker, you had to go in and put yourself at risk. And I thought, ‘Well, that’s not screaming, “We value you.”’ Actually, if you’re key worker, you’ve gotta go in, whereas everybody else sat at home. (Relative 1)

Many ancillary staff felt included in the Clap for Carers (later Clap for Heroes), an initiative introduced during the first lockdown in the UK in which the public was encouraged to clap out of a window or from a balcony as a demonstration of gratitude towards key workers during the pandemic. However, many of the ancillary staff participants felt ambivalent about the recognition, appreciating the gesture but also feeling the recognition soon faded after people became accustomed to the ‘new normal’.

Managers’ recognition of growing work demands was valued by ancillary staff:

[In order to train and support us] we got sent some videos about good hygiene and how to maintain good hygiene. We’re given a little longer breaks, so that we’re able to focus more. We get more contact with management, who are just keen to do spot checks. And that helps. Identify anything that they’re not happy with, so it could be fixed quite quickly. It makes you feel more safe. (Kitchen assistant 1)

## Discussion

Participants in our study recounted generally positive stories of positive working environments, staff team cohesion, and good management and leadership. Only one participant reported negative experiences with their employer. The contributions and experiences of ancillary staff during the pandemic were extensive with many taking on different and/or extra workloads, demonstrating a sense of personal responsibility towards residents, and being flexible around their own family and caring responsibilities.

Our findings highlight the importance of support, recognition, job satisfaction and teamwork for this workforce. This has implications for practice and policy.

### Support systems

The regulations around cleaning, PPE and testing increased the sense of safety and wellbeing for many ancillary staff participants, while at the same time increasing their workload.^14,15^ Other studies have reported staff being frustrated because of the time-consuming process of and the lack of monetary compensation for regularly testing, especially in countries without free tests.^16^ In our study, care home managers, ancillary workers, care home residents and their relatives appreciated government assistance, but often saw it as late, not adequately reflecting the care home sector (with its varied layouts, residents and staff), and more frustrating than helpful. Additionally, managers in our study reiterated previously reported feelings of limited understanding of care homes by government when issuing guidance.^6^ The pandemic laid bare a pre-existing ignorance relating to social care by the government, and care homes were hit hard by this.^17^

Support from care home managers and colleagues was valued by most ancillary staff participants in this study, such as through enhanced communications, events and tokens of appreciation. Casual support groups, such as WhatsApp, were widely accessed means of quick support for care home managers and staff^18,19^ and may signal increasing acceptability of technology in this sector.

There are different ‘coping mechanisms’ at play that may explain the difference in our ancillary staff participants’ general wellbeing and that of other staff. A survey of care home nurses and managers found most (80%) of these participants reported very negative experiences, including not being valued, poor terms and conditions of employment, being blamed for deaths and being pressured to transport residents with unknown COVID-19 status.^20^ There were some comforting prospects, however, including an acknowledgement of the skill, dedication, professionalism of those working in the care home sector. ^20^ The largely positive experiences in our ancillary work sample might be counter-intuitive, considering the high pressure on care home staff during the pandemic. However, this positive picture of intra-home support is reflected in other studies.^21^

### Recognition

The data indicate that emotional and tangible recognition from colleagues and managers enhance the wellbeing and motivation of ancillary staff. Recognition may take a material form, such as presents, food and drinks, and salary or bonuses, as well as verbal expressions of gratitude and awards to show appreciation. McBride and Martínez Lucio^22^ investigated the way people perceive and judge skills and contributions in relation to certain types of work and focused on the work of cleaners in public sector organisations (not social care), who are often perceived as ‘unskilled’ workers. Our study captured the views of this workforce of their skills both prior to and during the pandemic, which may be similar to the position of others in the ‘invisible workforce’ of cleaning and catering in health care settings, such as maternity units.^23^ Perception of cleaning as ‘dirty work’ potentially adds to stigma and degrades the status of the worker.^24^ However, in our study the cleaners and other ancillary staff interviewed were not working in isolation, and while rarely seen by ‘the public’, they were appreciated by colleagues and residents, and seemed to be content with their experiences and contributions. This contentment was largely reflected in participants’ responses to the ‘Clap for Carers’ activity in 2020 in the UK to show support to health care workers, subsequently expanded to include other key workers. As reflected in other studies,^25^ ancillary workers in our study had an ambivalent attitude towards this initiative. Some appreciated the applause, while others felt excluded or deemed it a ‘hollow gesture’.

### Job satisfaction

Studies suggest that cleaners are often happy in their jobs, possibly reflecting the generally low expectations they had of the work when they were first employed.^26,27^ Our study confirmed high levels of satisfaction with work, but this was due more to the galvanising impact of the pandemic on the contribution they felt able to make to offer care and save lives. Satisfaction with work among the wider group of ancillary staff interviewed came from teamwork, a sense of support, feelings of commitment and responsibility towards one another and to residents. The pandemic created a sense of urgency through crisis which brought many care home staff, including ancillary workers, closer together and enhanced resident contact.^28^ Furthermore, care homes’ isolation following lockdowns and their enhanced continued isolation created a bubble in which many staff felt determined to protect both themselves and care home residents; a sense of ‘doing what they had to’. The abovementioned recognition through positive appraisal and emotional support functioned as a coping mechanism^29^ that seemed to make the job manageable for the ancillary workforce in this study. To maintain these coping mechanisms, proper policies and appropriate measures need to be in place in the social care sector. Other studies have pointed out that the social care sector needs to become less burdensome to ensure the wellbeing and retention of its workforce.^29^ This study demonstrates that it is essential to include the ancillary workforce in such policies.

### Teamwork

Leadership and management could provide the support described above, as well as create opportunities for ancillary staff around progression. Some care homes offered training to ancillary staff on PPE and enhanced cleaning procedures, while others trained ancillary staff to support residents with eating and drinking and personal care tasks. The pandemic has triggered role expansion in care services for older people,^30^ also evident in our study findings. Managers welcomed such changes and saw the increased potential for ancillary staff to move to direct care roles.

## Limitations

There were three main limitations with this study. First, our sample of participants did not include all sources of ancillary staff. While the composition of participants demonstrated diversity in terms of gender, ethnicity and age, we did not interview participants who worked on zero-hour contracts or were working in a care home via a private cleaning or catering company. The research team attempted to recruit such staff but was unsuccessful. This may have been due to the pressures of the pandemic on such agencies or the relatively short time span in which the research had to be carried out. The team also did not recruit undocumented migrants, even though domestic work is one of the main sectors where they would find work.^6^ We may have encountered different narratives if we had recruited a wider range of ancillary staff.

Second, we did not ask about remuneration. This was because we determined that it may have been difficult to obtain such information during the pandemic, when hours of work were variable. This means future research on overtime pay, bonus payments, pay progression by skill or experience and job intentions would be useful.

Third, we collected no details on employers’ practices around risk assessments for staff deployment. Future research on this would add further context, especially if they take account of new or different cleaning products as part of occupational health.

## Conclusions

Our research highlights how ancillary staff are an integral part of care home life and suggests that their contributions to residents should be more broadly recognised. During the pandemic, much of this became more apparent, as job roles expanded. This study demonstrates that including the ancillary workforce in policies around disaster planning, infection control, and mental health policies is essential for the wellbeing and retention of this workforce. Teamwork was an important source of support for ancillary staff, as well as feeling valued and recognised, both amplified due to the pressure and isolation affecting care homes during the lockdowns. Recognising and acknowledging the contributions made by ancillary workers is one step towards providing the appropriate support and dynamic leadership this workforce can benefit from to continue to provide care to care home residents.

## Supplemental Material

Supplemental Material - ‘I wasn’t on the front line *per se*, but I was part of health care’: Contributions and experiences of ancillary staff in care homes in England during the COVID-19 pandemic Supplemental Material for ‘I wasn’t on the front line *per se*, but I was part of health care’: Contributions and experiences of ancillary staff in care homes in England during the COVID-19 pandemic by Olivia Luijnenburg, Kritika Samsi, Ian Kessler, Caroline Norrie, Stephen Martineau and Jill Manthorpe in Journal of Health Services Research & Policy.
