# Supplementary material for: ‘I wasn’t on the front line per se, but I was part of health care’: Contributions and experiences of ancillary staff in care homes in England during the COVID-19 pandemic
Source: J Health Serv Res Policy. 2024 Apr 20;29(3):143–52. doi: 10.1177/13558196241246178 (PMC11151701; doi:10.1177/13558196241246178)
Supplement: Supplemental Material - ‘I wasn’t on the front line per se, but I was part of health care’: Contributions and experiences of ancillary staff in care homes in England during the COVID-19 pandemic [file sj-pdf-1-hsr-10.1177_13558196241246178.pdf]

Online supplement:

Excerpt of table stakeholder workshops and co-creation of final outputs in this study

Stakeholder suggestions	Answers and actions
Add CQC stakeholder to workshop.	We invited [name] from CQC and she joined for the second stakeholder workshop.
Make sure to situate the study 'in the pandemic' (for example, when we conducted the study, the vaccination had not yet been made mandatory in care homes).	We will include this in the background and context of our study reporting.
Stakeholders wondered if the positivity of the recruited ancillary staff reflected the greater national picture well.	We acknowledge the bias in our sample and will make sure to report this in our outputs.<br>On the other hand, it will hopefully feed into the 'good practice model' and inform homes in which ancillary staff were less positive.
In the carers' group, a lot of people did not feel aware of what happened in a loved one's care home during Covid. They would have appreciated information about what ancillary staff were doing to support their loved ones.	We will include how important it is to communicate the contributions of ancillary staff to carers and family and friends.
The idea of 'co-creation' is overly used and we need to state well what we mean by that. It would be good to see the integration of the team in a more general sense (ancillary staff being integrate din the decision making and larger care home team), as well as in this research (integrating their suggestions and views directly into the outcomes).	This table reflects how the final model is 'co-created'. Furthermore, the qualitative data (quotes) will be integrated into the final outputs of the research. This is the strength of qualitative methodologies; the direct words and suggestions of participants are incorporated in the findings and outcomes.
It was suggested to incorporate more infographics in the final model as managers will not have time to read lots. Als looking at "we found that this worked" is a good starting point for recommendations.	This will be considered for the final output.
From a regulators point of view, this study is a good reminder; "do we pick up enough of these voices in our reviews?" Anything that puts these stories out there (not even in an implementable form) is very useful.	We will disseminate as widely as possible and will be very happy if our invited stakeholders pass it on to anyone they think might be interested.
